# Investigation of Non-Coding RNA-Related Autophagy Alterations in Drug-Resistant Multiple Myeloma Plasma Cells 

**DOI:** 10.30699/ijp.2024.2022061.3256

**Published:** 2024-10-02

**Authors:** Leila Sarafraznia, Zari Tahan Nejad Asadi, Dian Dayer, Mohammad Ali Jalalifar, Nariman Ghanatir

**Affiliations:** 1 *Department of Laboratory Sciences, School of Allied Medical Sciences, Ahvaz Jundishapur University of Medical Sciences, Ahvaz, Iran*; 2 *Thalassemia & Hemoglobinopathy Research center, Health research institute, Ahvaz Jundishapur University of Medical Sciences, Ahvaz, Iran*; 3 *Cellular and Molecular Research Center, Medical Basic Sciences Research Institute, Ahvaz Jundishapur University of Medical Sciences, Ahvaz, Iran*

**Keywords:** Multiple myeloma, Drug resistance, Autophagy, non-coding RNA, Plasma cell

## Abstract

**Background & Objective:**

Multiple myeloma (MM) drug resistance is thought to be caused by the development of protective autophagy. This work aimed to assess the non-coding RNA (ncRNA) autophagy-related alterations in drug-resistant (DR) myeloma cells.

**Methods:**

DR Plasma cells were extracted from the bone marrow of DR patients referred to Baghai 2 Hospital in Ahvaz, Iran. The cells were grown in RPMI-1640 media containing 10% FBS and 1% Pen/Strep and incubated at 37˚C and 5% CO_2_. After six passages, the plasma cells were precisely isolated and utilized as DR cells. The U266B1 cell line (IBRC C10148) was grown in the RPMI-1640 media containing 10% FBS and 1% Pen/Strep and utilized as drug-sensitive (DS) cells. The relative expression of the genes was determined using the Real-time PCR method. Statistical analysis of the data was performed using GraphPad Prism 8 software.

**Results:**

When the DR cells were compared to the DS cells, there was a notable increase in the expression of *ULK1* and *LC3B*. However, expression of *P62* in the DR plasma cells showed a significant decrease compared to the DS plasma cells. The *miR-1297* level was considerably higher in the DR cells than in the DS cells. Although, there was no statistically significant difference in the expression of *miR-26a-5p* between the DS and DR cells. The DR cells exhibited a statistically significant increase in the expression of *MALAT1* and *SNHG6*.

**Conclusion:**

Drug resistance in MM cells may result from overexpression of non-coding RNAs involved in autophagy.

## Introduction

Multiple myeloma (MM) is a malignant plasma cell disease that accounts for 1.7% of all cancer cases in the world today (1). MM accounts for around 13% of hematological malignancies (2). Some choices for MM treatment are immunotherapy, CAR-T cell therapy, chemotherapy, corticosteroids, bone marrow transplantation, and radiation therapy (3). Chemoresistance causes an enormous number of MM patients to remain incurable (4). Examining various drug resistance pathways is a beneficial way to increase the efficacy of therapeutic approaches (4). Proteases have an important function in the context of MM (5). Proteases alter the bone marrow microenvironment and have a role in tumor growth and metastasis (6). Several studies have shown that proteases have a role in drug resistance in cancer (7). Proteases have the potential to alter the expression of drug efflux pump-related proteins or proteins involved in the degradation of chemotherapeutic drugs (8). In this regard, protease inhibitors (PIs) have been investigated as potential treatment in MM (9). The 26S is a well-known proteasome in the MM pathophysiology (10). Bortezomib is a powerful PI that inhibits the β1 and β5 subunits of the 26S proteasome (11). According to previous studies, a large number of patients with MM exhibit PI resistance (12). Research demonstrates that long non-coding RNAs (LncRNAs) induce PI resistance (13). LncRNAs can act as microRNA sponges. Sponging prevents microRNAs from inhibiting target carcinogenic gene expression (14). The sponging of microRNAs by LncRNAs is the initiator of a series of signaling pathways that lead to angiogenesis, cancer development, and metastasis (15). Autophagy is thought to be one significant process that leads to resistance in MM (16). Consequently, looking into the role of non-coding RNAs in DR is advantageous in MM treatment (17). The human *miR-26* family consists of the *miR-26a*, *miR-26b*, *miR-1297*, and *miR-4465*(18). This family regulates drug resistance, invasion, and metastasis and serves as a tumor suppressor or generator (19). Previous data suggests that *miR-26a-5p* and *miR-1297* affect the expression of the *ULK1* (a key regulator of autophagy) expression (20). It has been shown that autophagy prevents cell death over an extended therapy duration and compensates for proteasome failure (21). Research findings indicate that the bortezomib-resistant myeloma cells had elevated levels of AMPK in comparison to the bortezomib-sensitive cells (22). The bortezomib-resistant myeloma cell lines have been shown to express *LC3B* at high levels (23). Previous research indicates that *LC3B *inhibition results in drug sensitivity (24). Furthermore, *P62* encourages myeloma cell survival and the emergence of treatment resistance (25). Certain LncRNAs have been investigated as potential biomarkers for MM (26). *MALAT1* is a well-known and conserved long noncoding RNA that plays an important role in numerous biological processes (27). *MALAT1* is strongly expressed in several cancers, such as stomach, colon, ovarian, chronic myeloid leukemia, and myeloma (28). Many studies have identified a link between *MALAT1* and an unfavorable outcome in cancer. It has been documented that chemotherapy increases *MALAT1 *expression (29). According to the Xu *et al.* study, exosomal *MALAT1* absorbs *miR-26a/26b* to increase PI3K/Akt pathway activity in colorectal cancer (29). Also, there have been reports of altered *SNHG6* expression levels in MM (30). Certain studies show that *SNHG6* has an improving impact on Bcl2 expression and apoptosis suppression (31). In CRC cells, *SNHG6* knockdown suppresses migration, invasion, and epithelial-mesenchymal transition (EMT) (32). *SNHG6* has a negative feedback loop on *miR-26a* expression (20). A recent study found that *SNHG6* promotes protective autophagy through the *miR-26a-5p*/*ULK1* axis (20). Some evidence, however, contradicts the concept of microRNA involvement in carcinogenic gene suppression. According to one study, *miR-26a* inhibits autophagy in Doxorubicin-treated HepG2 cancer cells by suppressing *ULK1*(33)*. *Yang *et al.* discovered a *Meg3-miR-1297* connection in testicular germ cell tumors that promoted PTEN/PI3K/AKT pathway activation and cancer cell proliferation (34). Given the contradicting hypotheses about the algorithm of change of ncRNA expression in chemotherapy resistance in cancer, the current study aimed to investigate the changes in ncRNAs and autophagy-related genes in myeloma cells from persons with MM who are resistant to chemotherapy.

## Materials

### Study Design

A case-control study was carried out. The DS U266B1 myeloma cell line served as the control group. The case groups were made up of DR plasma cells obtained from individuals with MM. The expression of autophagy-related genes and ncRNAs was evaluated using real-time PCR analysis. 

### Preparation of Drug-Sensitive Cell Line

The U266B1 myeloma cell line (IBRC C10148) was purchased from the Iranian Biological Resource Center and grown in the RPMI-1640 media (Sigma, USA) containing 10% FBS (Sigma, USA) and 1% Pen/Strep (Sigma, USA) and utilized as drug-sensitive (DS) cells. 

### Patients

DR plasma cells were obtained from three individuals with MM referred to Baghai 2 Hospital, Ahvaz, Iran. An expert physician validated the presence of chemotherapy resistance. All participants signed an informed consent form. Table 1 contains patient information.

### Inclusion Criteria

Inclusion criteria were the existence of an M peak on serum and urine electrophoresis, as well as persistent MM clinical symptoms and a large number of plasma cells following chemotherapy. 

### Exclusion Criteria

Patients who suffered from cardiovascular disease, ALL, AML, thalassemia, and cyclic anemia were excluded from the study. 

### Preparation of Drug-Resistant Cell Line

The mononuclear cells were purified from patients' bone marrow aspirate by Faycol (Sigma, USA). Based on the manufacturer's recommendation, the CD138 + plasma cells were isolated using a human CD138 positive magnetic beads isolation kit (Genekam Biotechnology, Germany). The purity of each fraction was determined using an anti-CD138 antibody (ab128936) (Abcam, USA)) in BD FACS Aria II Sorter flow cytometer (Iran). The CD138 + plasma cells were maintained in RPMI-1640 (Sigma, USA) containing 10% fetal bovine serum (FBS) (Sigma, USA) and 1% Pen/strep (Sigma, USA) at 37˚C with 5% CO_2_. The culture medium was exchanged every 2 days. The unspecific cells were eliminated after six generations of alternate passages. The specific plasma cells were validated using Giemsa staining. 

### RNA Extraction and cDNA Synthesis

According to the manufacturer's recommendations, RNA was extracted using an RNA extraction kit (AnaCell, Iran). The purity of RNA samples was determined using a nanodrop (Thermo, Canada). OD260/OD280 ratios between 1.8 and 2 indicated an acceptable quality of RNA. According to the manufacturer's recommendations, cDNA synthesis was carried out in 20 μL volume using a DNA synthesis kit (AnaCell, Iran). 

### Real-time qPCR

The specific primers were designed using the Primer3 software version 4.1.1. Table 2 displays the sequence of the primers. Real-time qPCR was carried out in a 20 µL reaction using a Quant Studio 3 Applied Biosystems Real-Time PCR System Thermal Cycler (Thermo Fisher Scientific®, UK). The reaction contained 10 µL of Master Mix (Ampliqon, Denmark), 0.8 µL of each primer (10 µmol/L), 2 µL of cDNA template (~100 ng), and 6.4 µL DNase-free distilled water. The PCR settings included an initial denaturation at 95˚C for 15 min, followed by 40 cycles of denaturation at 95˚C for 30 s, annealing, and extension at 60˚C for 60 s. U6 was used as an internal control for ncRNAs. Other genes were normalized according to GAPDH. Every experiment was run in triplicate. Two distinct reactions free of cDNA or RNA were conducted concurrently as negative controls. Data were analyzed using the 2^-ΔΔCt^ method.

### Statistical Analysis

GraphPad Prism Version 8.4.3 software was used to analyze the data. The normality of the data was examined using the Shapiro-Wilk statistical test. The parametric t-test (t-test) was used for statistical analysis. Data were presented as Mean±SD. Significant differences between case and control groups were reported as follows: #,*P<0.05, ##, **P<0.01, ###, *** *P*<0.001, **** *P*<0.0001.

## Results

### Expression of the Autophagy-related Genes

*ULK1* expression

**Fig. 1:**
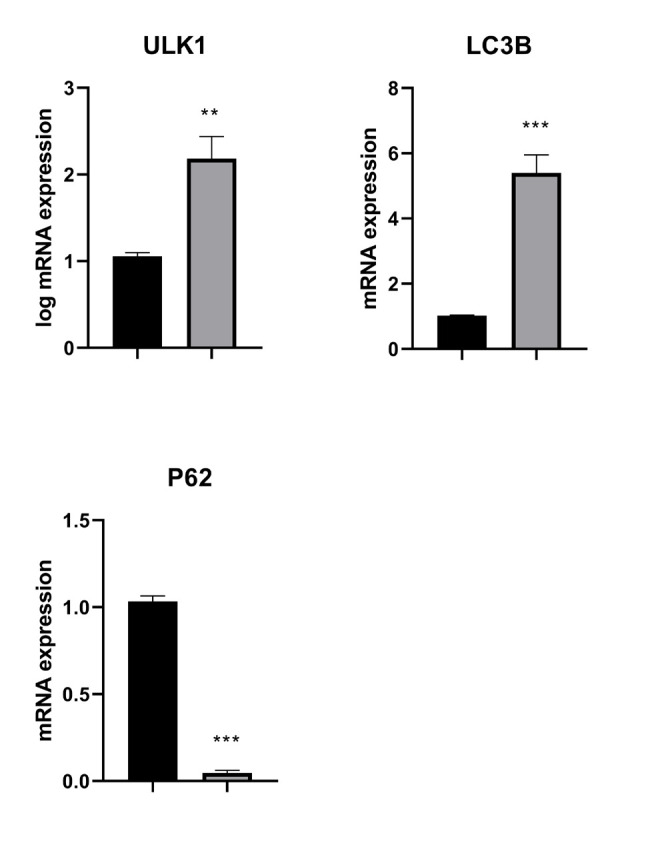
Autophagy-related gene expression in the DS U266B1 and DR myeloma cells. The expression of ULK1, LC3B, and P62 in the DS U266B1 myeloma cells and DR plasma cells derived from the chemotherapy-resistant patients suffered from MM. GAPDH was used as a housekeeping gene. Results are means ± SD for three independent experiments with duplicated wells.**,***, represent the significant difference between groups at *P*< 0.01, *P*< 0.001, respectively.

According to the data analysis, *ULK1* expression was considerably higher in DR cells than in DS U266B1 cells (*P*<0.01) (Figure 1).

*LC3B *Expression

Expression of *LC3B* was substantially higher in the DR cells than in DS U266B1 cells (*P*< 0.001) (Figure 1).

*P62 *Expression

The DR cells exhibited substantially lower levels of *P62* than DS U266B1 cells (*P*< 0.001) (Figure 1).

### Autophagy-related MicroRNAs Expression

*miR-26a-5p*

**Fig. 2:**
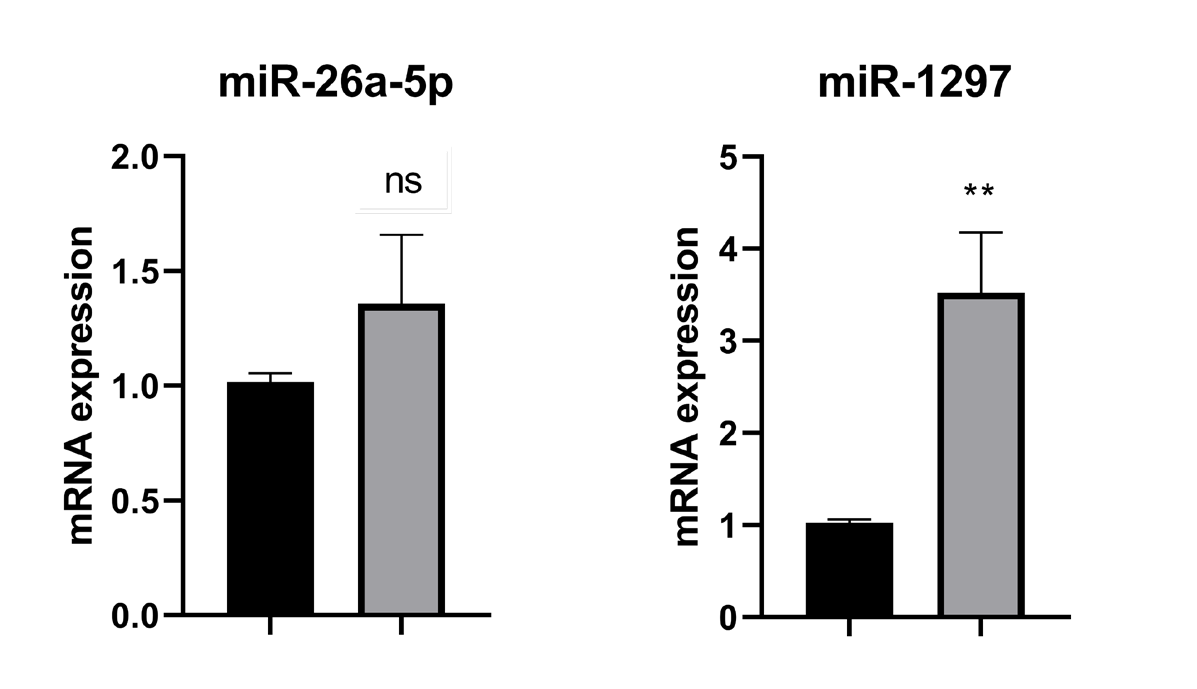
Autophagy-related MicroRNA expression in the DS U266B1 and DR myeloma cells

The study results found no evidence of a significant difference in *miR-26a-5p* gene expression level between the DS and DR U266B1 cells (*P*=0.1141) (Figure 2).

*miR-1297*

*MiR-1297* expression was considerably higher in the DR cells than in DS U266B1 cells (*P*=0.0002) (Figure 2).

### Autophagy-related LncRNA Expression

*MALAT1*

**Fig. 3:**
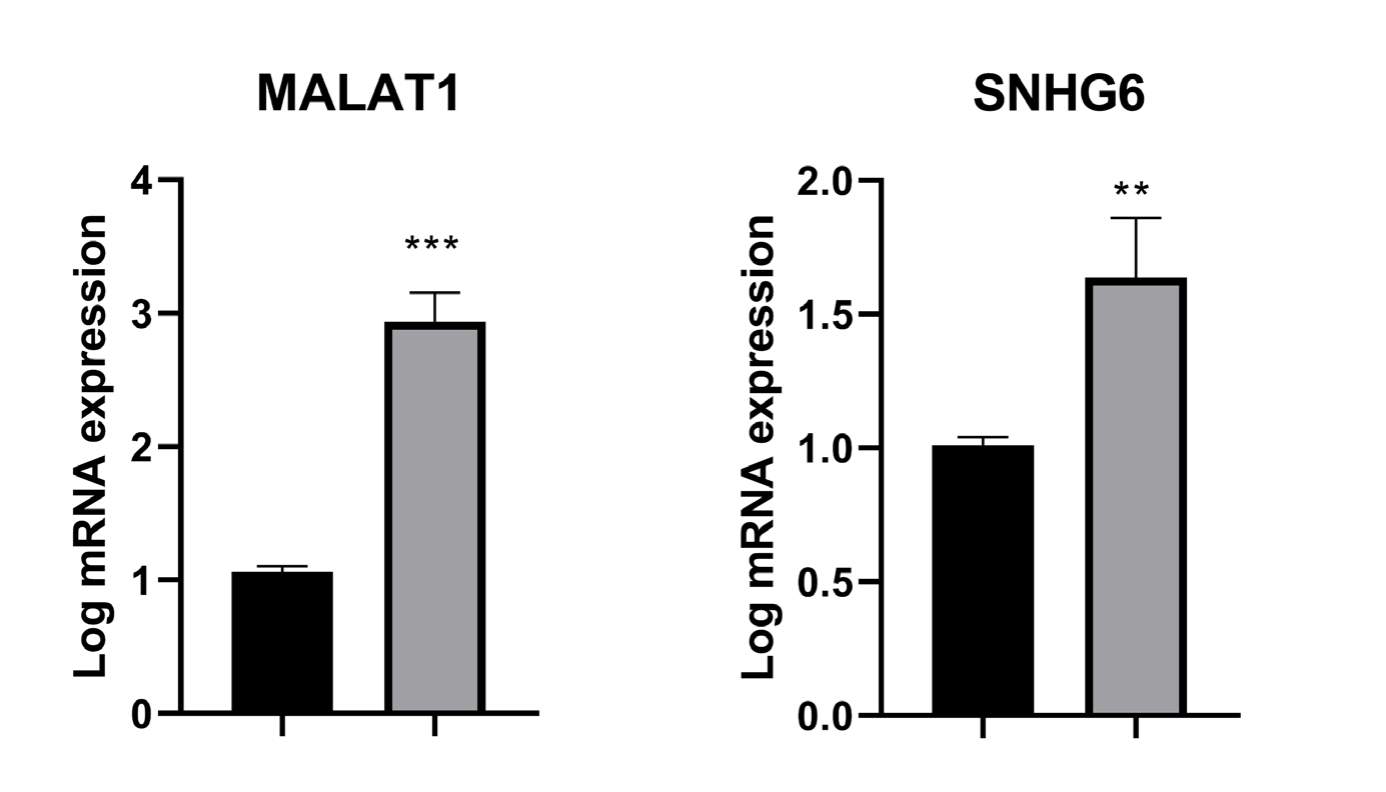
Autophagy-related LncRNA expression in the DS U266B1 and DR myeloma cells

The data analysis revealed a significant increase in the expression level of the *MALAT1 *in the DR cells compared to DS U266B1 cells (*P*<0.001) (Figure 3).

*SNHG6*

The data analysis revealed that the expression level of the *SNHG6* gene was considerably greater in the DR cells compared to DS U266B1 cells (*P*<0.01) (Figure 3).

## Discussion

Despite significant technological improvements, MM remains an incurable cancer (35). Drug resistance is one of the major challenges in the treatment of MM. Meanwhile, the mechanisms underlying drug resistance in MM remain unknown (35, 36). Recent research has demonstrated the role of cytogenetic and epigenetic changes on medication resistance (37). Some studies found a 17p13 deletion in Bortezomib-resistant patients (12). Furthermore, patients with higher unmethylated DNA demonstrated reduced survival following bortezomib treatment (38). Meanwhile, NFkB methylation was linked to a worse survival rate among MM patients (39). In addition, the dysregulation of signaling pathways contributes significantly to drug resistance (40). The alterations in the bone marrow microenvironment play a key role in MM treatment resistance (41). Recent investigations found a noticeable rise in IGF-I/IGF-IR among bortezomib-resistant individuals (42). Research shows that the downregulation of P-gp, the product of the MDR1 gene, leads to DR in MM (43). Some evidence suggests that P53 suppression and NFKB overexpression play an important role in bortezomibe resistance (44). Protective autophagy is one of the most significant processes in drug resistance of hematologic malignancies (16). Previous studies have shown that the active autophagy pathway is essential for cancer cell survival. Bortezomib is a common medication used to treat MM (45). The investigation by Lernia *et al.* showed that bortezomib therapy results in autophagy suppression in MM. The researchers found that combining therapy with bortezomib and hydroxychloroquine downregulates LC3b and P62 expression in plasma cells from MM patients (23). The study by Jiang et al. revealed a significant apoptosis induction following combination therapy of bortezomib and autophagy inhibition in AML cells (46). A clinical trial investigation looked at the concurrent impact of autophagic and proteasome pathways on MM treatment. The study's findings demonstrated that, in individuals resistant to bortezomib, targeting autophagy with hydroxychloroquine improved the effects of myeloma treatment (47). In this regard, we investigated the changes in autophagy-related gene expression in DR plasma cells compared to the DS U266 plasma cell line. U266 cells may not accurately reflect the variety of patient-derived plasma cells (48). However, Ndacayisaba *et al.* found that U266 cells were CD138+CD56+CD45- and had a similar morphology to MM Plasma cells (49). Our survey revealed that the resistant group's *ULK1 *expression was significantly higher than the sensitive cells. The study by Tang *et al.* revealed that *ULK1 *expression reduction caused apoptosis and cisplatin sensitivity in NSCLC cells (50). Bhattacharya et al reported impaired leukemic cell homing among *ULK1*-deficient mouse models with AML (51). According to some research, autophagy inhibition results in cancer cell death (52). Furthermore, our study exhibited an inevitable increase in *LC3B* gene expression in the DR group. *LC3B* is another crucial gene in the autophagy process (53). Bortezomib has been shown to increase the expression of Beclin-1, LC3-I, and LC3-II in hepatocellular carcinoma (54). Frassanito *et al.*'s work on myeloma cell lines and cancer fibroblasts derived from a chemotherapy-resistant patient identified bortezomib as a protective autophagy activator that acts by increasing LC3-II levels and decreasing *P62* levels (55). Milan *et al.* reported that *P62*-dependent autophagy leads to tolerance to proteasome inhibitors (56). They found that SQSTM1/p62 suppression alleviates proteasome inhibitor resistance in MM plasma cells (56). According to Riz *et al.* research, *P62* suppression leads to susceptibility to proteasome inhibitor medicines (57). However, the current investigation found that the DR group had lower levels of P62 expression than the DS group. In our study, the expression of *MiR-26a-5p* in DR and DS cells did not differ considerably. In the Hu *et al.* survey, plasma cells from MM patients exhibited lower levels of *miR-26a* expression than controls (58). The researchers also found a link between miR-26a overexpression and improved therapeutic efficacy of bortezomib and melphalan Jung identified that patients with resistant myeloma who did not respond to lenalidomide, and dexamethasone had lower expression of *miR-26a* (58)*. *According to Wang et al. research, a decrease in the expression of *miR-26a-5p* in colorectal cancer cell lines led to an increase in treatment resistance (59). However, overexpression of *miR-26a-5p* resulted in tamoxifen resistance modification (59). According to our findings, the expression of *miR-1297 *by DR cells is significantly higher than DS cells. In a study conducted by Singh et al., the expression of *miR-1297* was found to be considerably higher in drug-resistant breast cancer. According to this research, the expression of *MALAT1 *was considerably higher in the DR group than in the DS cells (60). Similarly, Hu et al. work demonstrated increased expression of *MALAT1* in bortezomib-resistant myeloma cell lines (61). Handa *et al.*'s research found that MALAT1 plays a role in developing extramedullary myeloma after chemotherapy (62). Fu *et al.* found increased expression of *MALAT1* in 5-FU-resistant cancer cells (63). The DR group's SNHG6 expression in the current study was much higher than in the DS cells. Wang et al. discovered that raising the expression of *SNHG6* in colorectal cancer cells through external interventions resulted in medication resistance (59).

In total, a comparison of the findings of this study with previous data reveals that autophagy is an important biological mechanism for MM plasma cell adaptability and survival. Autophagy is necessary for the development of drug resistance in MM plasma cells. The study of autophagy-related ncRNAs and their regulators may aid in understanding the mechanism of drug resistance in MM. However, drug resistance in MM results from the combination of various factors and the mechanism of action of non-coding RNA varies depending on the type of cancer cell and chemotherapeutic drug treatments.

**Table 1:** Patient information

Dosage of bortezomib (mg/m^2^)	Gender	Age	Medications	No
1-1.5	Female	50	Bortezomib/dexamethasone/kytril/doxil/pantoprazole/sertraline/abitant	1
1-1.5	Male	73	Bortezomib/dexamethasone/kytril/endoxan	2
1-1.5	Male	49	Bortezomib/dexamethasone/endoxan/zometa	3

**Table 2:** Characteristics of the primers

Primer name	Primer sequence
GAPDH	F: GGAGCGAGATCCCTCCAAAAT
	R: GGCTGTTGTCATACTTCTCATGG
ULK1	F: CCTGCTGAGCCGAGAATG
	R: CTGCTTCACAGTGGACGACA
LC3B	F: CGATACAAGGGTGAGAAGCAG
	R: CTGAGATTGGTGTGGAGACG
P62	F: GCACCCCAATGTGATCTGC
	R: CGCTACACAAGTCGTAGTCTGG
MALAT1	F: ATGCGAGTTGTTCTCCGTCT
	R: TATCTGCGGTTTCCTCAAGC
SNHG6	F: CTCTGCGAGGTGCAAGAAAG
	R: AATACATGCCGCGTGATCCT
HK	F: GGATGTCCAAATGCCTGTTATG
	R: GTGCGGTCAAAGCGATAATG
miR-1297	F: TTCAAGTAATTCAGGTG SL: GTCGTACCAGTGCAGGGTCCGAGGTATTCGCACTGGATACGACGTTTCA	
miR-26a-5P	F: CAAGTAATCCAGGATAGG\ SL: GTCGTATCCAGTGCAGGGTCCGAGGTATTCGCACTGGATACGACAGCCTA	

The expression of *miR-26a-5p* and miR-1297 in the DS U266B1 myeloma cells and DR plasma cells derived from the chemotherapy-resistant patients suffered from MM. HK was used as a housekeeping gene. Results are means ± SD for three independent experiments with duplicated wells. ** represent the significant difference between groups at *P*<0.01.

The expression of *MALAT1* and *SNHG6* in the DS U266B1 myeloma cells and DR plasma cells derived from the chemotherapy-resistant patients suffered from MM. HK was used as a housekeeping gene. Results are means ± SD for three independent experiments with duplicated wells. **,***, represent the significant difference between groups at *P*<0.01 and *P*<0.001, respectively.

## Conclusion

Drug resistance may be related to autophagy development in MM plasma cells. Increased miR-1297 levels may be associated with a worse prognosis of DR MM. MALAT1 and SNHG6 may be involved in the development of MM drug resistance. However, it should be noted that autophagy-related gene expression is dependent on patient characteristics, disease stage, or past treatments. Hence, we propose autophagy suppression through miR-1297, MALAT1 and SNHG6 downregulation to improve DR MM treatment. Further studies are recommended to elucidate the exact mechanisms involved in the autophagy-related LncRNAs.
